# The complete mitochondrial genome of *Engyodontium album* and comparative analyses with Ascomycota mitogenomes

**DOI:** 10.1590/1678-4685-GMB-2016-0308

**Published:** 2017-10-23

**Authors:** Xiao-Long Yuan, Xin-Xin Mao, Xin-Min Liu, Sen Cheng, Peng Zhang, Zhong-Feng Zhang

**Affiliations:** 1 Tobacco Research Institute of Chinese Academy of Agricultural Sciences, Qingdao, China; 2 Shanghai Tobacco Group Company Limited, Shanghai, China

**Keywords:** Engyodontium album, mitochondrial genome, comparative analysis, phylogenetic analyses

## Abstract

*Engyodontium album* is a widespread pathogen that causes different kinds of dermatoses and respiratory tract diseases in humans and animals. In spite of its perniciousness, the basic genetic and molecular background of this species remains poorly understood. In this study, the mitochondrial genome sequence of *E. album* was determined using a high-throughput sequencing platform. The circular mitogenome was found to be 28,081 nucleotides in length and comprised of 17 protein-coding genes, 24 tRNA genes, and 2 rRNA genes. The nucleotide composition of the genome was A+T-biased (74.13%). Group-II introns were found in the *nad1*, *nad5*, and *cob* genes. The most frequently used codon of protein-coding genes was UAU. Isoleucine was identified as the most common amino acid, while proline was the least common amino acid in protein-coding genes. The gene-arrangement order is nearly the same when compared with other Ascomycota mitogenomes. Phylogenetic relationships based on the shared protein-coding genes revealed that *E. album* is closely related to the Cordycipitaceae family, with a high-confidence support value (100%). The availability of the mitogenome of *E. album* will shed light on the molecular systematic and genetic differentiation of this species.

## Introduction

The *Engyodontium album* fungus is a member of the Cordycipitaceae family and it characterized by cottony, white colonies that produce numerous dry, tiny conidia. Evidence suggests that *E. album* can infect a wide range of invertebrates and vertebrates with a cosmopolitan distribution, including arthropods, reptiles, birds, mammals, and humans (Zimmermann, 2007). Infections caused by *E. album* can induce mild to severe disease, including eczema vesiculosum (Hoog, 1972), granulomatous skin lesions, brain abscesses (Seeliger, 1983), and keratitis (McDonnell *et al.*, 1984). In addition, some patients are even infected without being directly exposed to this fungus, e.g., by using an *E. album* product bassianin (Tucker *et al.*, 2004). With the incidence of *E. album* infection increasing throughout the world, it is necessary to explore the molecular characteristics and phylogenetics of *E. album* for effective therapeutic strategies. Unfortunately, the taxonomy of *E. album* genus remains unsettled.

Mitochondria are responsible for cellular respiration and energy production in eukaryotic organisms (Henze and Martin, 2003). Mitochondrial DNA (mtDNA) is typically circular and has its own replication machinery that is usually regulated by the nuclear genome (Hu *et al.*, *2004*). Owing to their high mutation rates, small sizes, and lack of recombination, mtDNAs have been widely used as informative molecular markers for phylogenetic analyses and species identification (Botero-Castro *et al.*, 2013). Recently, mtDNA was also used for DNA barcoding to facilitate identification in the fields of population genetics, comparative genomics, and evolutionary genomics (Kurbalija Novicic *et al.*, 2015; Qiu *et al.*, 2013). The mitochondrial genomes of fungi have been used as genetic markers for identification and classification purposes (Beaudet *et al.*, 2013). In 1997, Canadian researchers defined the goals of the fungal mitochondrial genome project as being to analyze the genome structure, gene content, and evolution of gene expression in fungal mitochondria (Paquin *et al.*, 1997). Fungal mitochondrial genomes are closed, circular-DNA molecules with lengths ranging from 10 to 80 kb and encode a respiratory chain subunit gene, an ATP synthase complex subunit gene, and ribosomal RNA and tRNA genes (Paquin *et al.*, 1997). As of November, 2016, 339 fungal mitochondrial genomes had been deposited in the National Center for Biotechnology Information (NCBI) database. The mitochondrial genomes of *Heterakis gallinae* and *Heterakis beramporia* were amplified by Wang *et al.* (2016) to develop useful markers for their systematic- and population-genetics study. Liu *et al.* (2014) sequenced the complete mitochondrial genome of *Micrura ignea* and made comparisons with other nemertean mitogenomes. However, the complete mitochondrial genome sequence remains unavailable for the genus *Engyodontium*.

In this study, we completely sequenced the *E. album* mitogenome to characterize and classify it. We also analyzed the gene content and structure, as well as codon utilization associated with protein-coding genes (PCGs). Other fungal mitogenomes were comparatively analyzed to gain additional insights into their gene content, structure, organization, and phylogenetic relationships.

## Materials and Methods

### Sample collection and DNA extraction

*E. album* (strain: ATCC-56482), isolated from a human brain abscess causing death in a female patient (Seeliger, 1983), was purchased from BeiNa Biological Technology Co., Ltd. (Suzhou, China). The strain was cultured at 24 °C in ATCC 200 Yeast Mold Agar medium (BD 271120). Fungus samples were collected after washing twice with sterile water and then stored at −80 °C. Total genomic DNA was isolated from the spores and mycelium using the E.Z.N.A. Fungal DNA Kit (Omega), according to the manufacturer’s instructions. The integrity of the genomic DNA was checked on a 1% agarose gel, and the concentration was detected using a NanoDrop 2000 UV-Vis spectrophotometer (NanoDrop).

### Sequence assembly, annotation, and analysis

*E. album* mtDNA was sequenced using an Illumina HiSeq2000 instrument and assembled using SPAdes software, version 3.6.1 (Bankevich *et al.*, 2012). The Bandage 0.7.1 program was used to check the assembly path and confirm the *E. album* mtDNA formed a circular molecule (Wick *et al.*, 2015). Moreover, iterative mitochondrial baiting was used to further verify the accuracy of the sequence from head to tail. PCGs were annotated using NCBI’s ORF-finder program (https://www.ncbi.nlm.nih.gov/orffinder/). Analysis of tRNA genes was conducted with the tRNAscan-SE 1.21 Search Server (http://lowelab.ucsc.edu/tRNAscan-SE/) (Lowe and Eddy, 1997). Complete ribosomal RNA genes were identified by alignment with the *Lecanicillium saksenae* mitogenome (GenBank accession no. KT585676) through BLAST (http://blast.ncbi.nlm.nih.gov/Blast.cgi). The circular genome map was constructed using OGDRAW (http://ogdraw.mpimp-golm.mpg.de/cgi-bin/ogdraw.pl) (Lohse *et al.*, 2007). The codon-usage frequency for each amino acid was determined with CodonW (Peden, 2000). The complete sequence of *E. album* mtDNA was deposited in GenBank under accession no. KX061492. Comparative analyses of the nucleotide sequence of each PCG and ribosomal DNA genes were conducted for *Acremonium chrysogenum*, *Fusarium oxysporum*, *Hypocrea jecorina*, *L. saksenae,* and *Metacordyceps chlamydosporia*. Strand bias was characterized by determining AT skewing and GC skewing, calculated using the relationships (A%–T%)/(A%+T%) and (G%–C%)/(G%+C%), respectively. Mitochondrial genome sequences were compared using the Blast Ring Image Generator (BRIG; Tablizo and Lluisma, 2014), with *E. album* mtDNA serving as the reference sequence. To estimate the evolutionary-selection constraints on genes in the Hypocreales and Ascomycota taxa, common PCGs were chosen to calculate the ratio of nonsynonymous and synonymous changes (Ka/Ks). Codon alignments were performed before pairwise Ka, Ks, and Ka/Ks ratios were calculated using DnaSP software, version 5 (Librado and Rozas, 2009).

### Phylogenetic analysis

To determine the phylogenetic location of *E. album*, currently available complete or near-complete mitochondrial genomes of fungi were used for phylogenetic analysis. The clade including *Phaeosphaeria* nodorum and *Sporothrix schenckii* was set as the outgroup. A global analysis was performed using 13 shared PCGs (*nad1–nad6*, *nad4L*, *cox1–cox3*, *atp6*, *atp8*, and *atp9*) among *E. album* and other related mitochondrial genomes. These genes were individually aligned using the default settings of MAFFT (Katoh *et al.*, 2005), and then these 13 alignments were concatenated using CLUSTAL X software, version 1.81 (Thompson *et al.*, 2002). Finally, a phylogenetic tree was constructed using RAxML version 8.1.12 and MrBayes 3.2, using the general time-reversible model (Stamatakis, 2014; Huelsenbeck and Ronquist, 2001). For each node of the ML tree, bootstrap support was calculated using 1000 replicates. For the Bayesian tree, the initial 10% of values were discarded as burn-in and 4 simultaneous chains were run for 10,000,000 generations.

## Results

### Genome organization, structure, and composition

**Figure 1:**
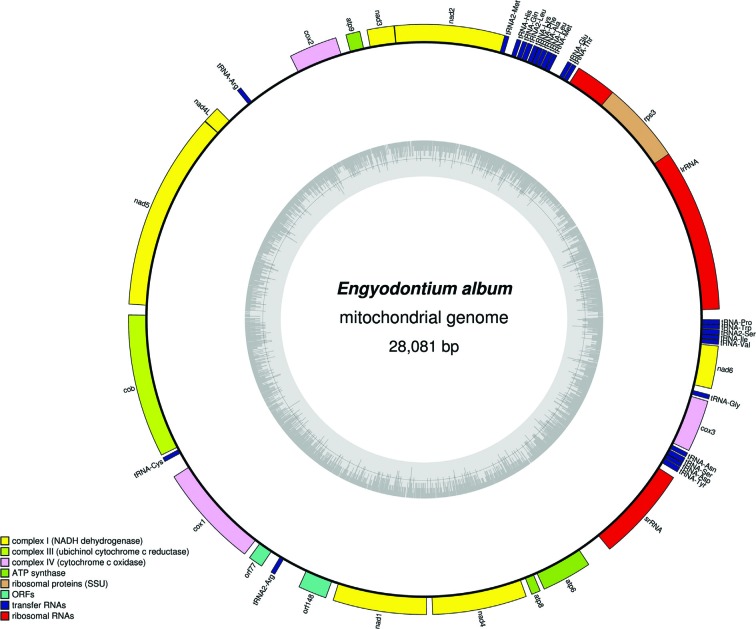
Mitochondrial genome map of *Engyodontium. album*. Genes are transcribed in a clockwise direction.

The complete mt genome of *E. album* is a circular molecule of 28,081 bp containing 17 PCGs, 24 transfer RNA genes, and 2 ribosomal RNA genes. All mt genes of *E. album* are transcribed in the same direction. The average base composition of the complete *E. album* mitogenome is 37.39% A, 14.65% C, 11.21% G, and 36.74% T. Therefore, the nucleotide composition of the *E. album* mt genome is biased toward A+T (74.14%). The composition of the *E. album* mt genome sequence was found to be strongly skewed away from A, in favor of T (AT skew = –0.01), and the GC skew was 0.14, as observed with those of other Cordycipitaceae family members. Moreover, Figure 1 shows that the mitogenome includes 24 tRNA genes and 2 rRNAs genes (large and small subunits).

### Protein-coding genes

The *E. album* mitochondrial genome encodes 17 proteins. Among these, seven protein-coding genes (PCGs) are involved in oxidative phosphorylation (*nad1*–*nad6*, *nad4L*), three genes encode different subunits of the cytochrome c oxidase complex (*cox1–cox3*), three genes encode different subunits of ATP synthase (*atp6*, *atp8*, and *atp9*), one gene encodes the cytochrome b subunit (*cob*), one gene encodes a ribosomal protein (*rps3*), and two genes encode open reading frames (ORFs), namely *ORF77* and *ORF148*. Group-II introns were found in the *nad1, nad5*, and *cob* genes. Moreover, all PCGs in the mt genome start with ATG, 13 genes (*nad2*, *nad3*, *nad4L*, *nad5*, *nad6*, *atp6*, *atp8*, *atp9*, *rps3*, *cox3*, *cob, ORF77*, and *ORF148*) use TAA as the termination codon, and four genes (*cox1*, *cox2*, *nad1*, and *nad4*) end with TAG (Table 1).

**Table 1:** List of annotated mitochondrial genes in *E. album.*

Gene	Position	Length (bp)	Start/stop codons	Anticodons
rrnL	154–2397, 4026–4559	2244		
rps3	2656–3930	1275	ATG/TAA	
tRNA-Thr [T]	4602–4672	71		TGT
tRNA-Glu [E]	4678–4750	73		TTC
tRNA-Met [M1]	4934–5006	73		CAT
tRNA-LeuUUN [L1]	5009–5090	82		TAA
tRNA-Ala [A]	5097–5168	72		TGC
tRNA-Phe [F]	5172–5244	73		GAA
tRNA-Lys [K]	5245–5317	73		TTT
tRNA-LeuCUN [L2]	5336–5418	83		TAG
tRNA-Gln [Q]	5426–5498	73		TTG
tRNA-His [H]	5520–5592	73		GTG
tRNA-Met [M2]	5713–5785	73		CAT
nad2	5778–7472	1695	ATG/TAA	
nad3	7473–7892	420	ATG/TAA	
atp9	7996–8220	225	ATG/TAA	
cox2	8371–9120	750	ATG/TAG	
tRNA-ArgCGN [R1]	10007–10077	71		ACG
nad4L	10481–10750	270	ATG/TAA	
nad5	10750–13813	3064	ATG/TAA	
cob	13968–16179	2212	ATG/TAA	
tRNA-Cys [C]	16219–16288	70		GCA
cox1	16548–18149	1602	ATG/TAG	
orf77	18234–18464	231	ATG/TAA	
tRNA-ArgAGN [R2]	18627–18697	71		TCT
orf148	19104–19550	447	ATG/TAA	
nad1	19659–21102	1444	ATG/TAG	
nad4	21187–22644	1458	ATG/TAG	
atp8	22725–22871	147	ATG/TAA	
atp6	22929–23708	780	ATG/TAA	
rrnS	24092–25559	1468		
tRNA-Tyr [Y]	25655–25739	85		GTA
tRNA-Asp [D]	25744–25816	73		GTC
tRNA-SerAGN [S1]	25818–25901	81		GCT
tRNA-Asn [N]	25916–25986	71		GTT
cox3	26020–26829	810	ATG/TAA	
tRNA-Gly [G]	26859–26930	72		TCC
nad6	27018–27683	666	ATG/TAA	
tRNA-Val [V]	27701–27772	72		TAC
tRNA-Ile [I]	27774–27845	72		GAT
tRNA-SerUCN [S2]	27847–27931	85		TGA
tRNA-Trp [W]	27936–28007	72		TCA
tRNA-Pro [P]	28009–28081	73		TGG

The relative synonymous codon usage (RSCU) value is a measure of the synonymous codons present in a coding sequence. If there is no codon-usage bias, the RSCU values equal 1.00. A codon that is used less frequently than expected will have an RSCU value of < 1.00, whereas a codon used more frequently than expected will have an RSCU value of > 1.00 (Sharp *et al.*, 1986). The results from the *E. album* mitogenome indicated that almost all amino acids (except for Met) showed codon-usage bias. The most frequently used codon in PCGs was UAU, followed by AUU and UAA, which is consistent with the (A+T)-rich content of the *E. album* mitogenome. CGC was the least used codon. Ile is the most commonly encoded amino acid in the *E. album* mitogenome, while Pro is the least common (Table 2).

**Table 2:** Number of codons and codon usages in mt protein-coding genes of *E. album.*

Amino acid	Codon	N	RSCU	Amino acid	Codon	N	RSCU
Phe [F]	UUU	462	1.55	Tyr [Y]	UAU	624	1.61
	UUC	135	0.45		UAC	150	0.39
Leu-UUN [L]	UUA	475	2.61	Ter [end]	UAA	482	1.61
	UUG	136	0.75		UAG	262	0.88
Leu-CUN [L]	CUU	182	1.00		UGA	154	0.51
	CUC	45	0.25	His [H]	CAU	137	1.57
	CUA	174	0.96		CAC	37	0.43
	CUG	81	0.44	Gln [Q]	CAA	104	1.13
Ile [I]	AUU	570	1.43		CAG	80	0.87
	AUC	150	0.38	Asn [N]	AAU	442	1.53
	AUA	474	1.19		AAC	137	0.47
Met [M]	AUG	160	1.00	Lys [K]	AAA	469	1.38
Val [V]	GUU	160	1.42		AAG	213	0.62
	GUC	42	0.37	Asp [D]	GAU	146	1.62
	GUA	183	1.63		GAC	34	0.38
	GUG	65	0.58	Glu [E]	GAA	159	1.33
Ser-UCN [S]	UCU	124	1.22		GAG	81	0.68
	UCC	73	0.72	Cys [C]	UGU	139	1.17
	UCA	110	1.08		UGC	99	0.83
	UCG	37	0.36	Trp [W]	UGG	107	1.00
Pro [P]	CCU	43	1.51	Arg-CGN [R]	CGU	33	0.47
	CCC	18	0.63		CGC	12	0.17
	CCA	33	1.16		CGA	40	0.57
	CCG	20	0.70		CGG	26	0.37
Thr [T]	ACU	85	1.10	Arg-AGN [R]	AGA	169	2.39
	ACC	65	0.84		AGG	144	2.04
	ACA	121	1.56	Ser-AGN [S]	AGU	151	1.48
	ACG	39	0.50		AGC	116	1.14
Ala [A]	GCU	67	1.72	Gly [G]	GGU	71	1.46
	GCC	22	0.56		GGC	25	0.51
	GCA	47	1.21		GGA	62	1.27
	GCG	20	0.51		GGG	37	0.76

N: number of codons. RSCU: relative synonymous codon usage

### Transfer and ribosomal RNA genes

**Figure 2:**
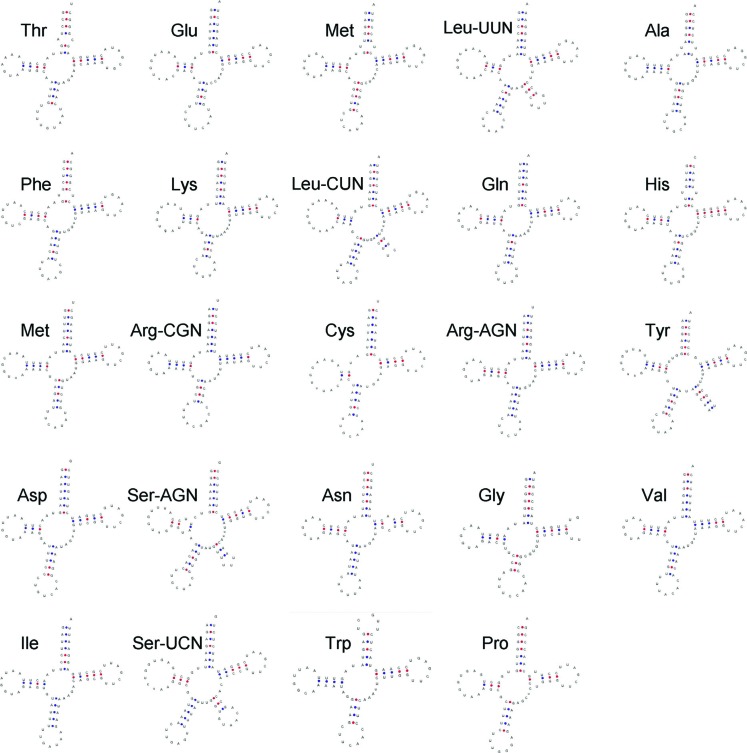
Predicted tRNA structures of *E. album*.

Twenty-four tRNAs were recognized in the mt genome of *E. album*, were interspersed between the rRNA- and PCGs, and ranged from 70 to 85 bp in length. Of these tRNAs, two forms each were identified for tRNA-Arg (AGN and CGN), tRNA-Ser (UCN and AGN), and tRNA-Leu (UUN and CUN). Taking into account their relative proximities, the tRNA genes could be considered to cluster into three groups: TEMLAFKLQHM (*trnT-TGT, trnE-TTC trnM1-CAT, trnL1-TAA, trnA-TGC, trnF-GAA, trnK-TTT, trnL2-TAG, trnQ-TTG, trnH-GTG*, and *trnM2-CAT*), YDSN (*trnY-* *GTA, trnD-GTC, trnS1-GCT*, and *trnN-GTT*), and VISWP (*trnV-TAC,trnI-GAT, trnS2-TGA, trnW-TCA*, and *trnP-TGG*), with the exception of four trn genes (*trnR, trnL, trnR2*, and *trnC*) that were scattered as single genes throughout the mt genome. All 24 tRNA genes were predicted to have the typical cloverleaf structure, except for tRNA-Tyr (UAU), tRNA-Ser (UCN and AGN), and tRNA-Leu (UUN and CUN) (Figure 2). These five tRNAs adopt a special structure that is widely found in the Sordariomycetes class and is a common feature for Hypocreales species. The *E. album* rrnL (16S rRNA) gene is located between tRNA-Pro and *rps*, while rrnS (12S rRNA) is located between *atp6* and tRNA-Tyr. The lengths of the rrnS and rrnL genes are 1,468 bp and 2,244 bp, respectively, and their A+T contents are 65.46% and 67.98%, respectively.

### Comparative analysis with other mt genomes

To better understand the gene contents and structure of this species in the Hypocreales order, which consists of six families, the mt genomes from *L. saksenae* (Cordycipitaceae), *Fusarium oxysporum* (Nectriaceae), *Hypocrea jecorina* (Hypocreaceae), *Metacordyceps chlamydosporia* (Clavicipitaceae), and *Acremonium chrysogenum* (Hypocreales incertae sedis) were chosen for comparative analysis. The genomes were similar in size, with the exception of *F. oxysporum* (Table 3). The results showed that genome size ranged from 25 kb to 42 kb. The AT-skew values for these species were all negative, while the GC-skew values were positive. As shown in Table 3, the AT-skew value of *E. album* is fairly close to that of *M. chlamydosporia*.

**Table 3:** Composition and skewing in the mitochondrial genomes of Hypocreales.

Species	Size (bp)	A%	C%	G%	T%	AT skewing	GC skewing
Ach	27,266	35.9	11.0	15.5	37.6	–0.02	0.17
Eal	28,081	36.7	11.2	14.7	37.4	–0.01	0.14
Fox	33,396	34.3	14.2	16.8	34.7	–0.01	0.08
Hje	42,130	37.0	12.2	15.1	35.8	0.02	0.11
Lsa	25,919	36.5	11.6	14.9	37.0	–0.01	0.12
Mch	25,615	35.6	12.7	15.6	36.2	–0.01	0.10

Ach: *Acremonium chrysogenum*; Fox: *Fusarium oxysporum*; Hje: *Hypocrea jecorina*; Lsa: *Lecanicillium saksenae*; Mch: *Metacordyceps chlamydosporia*.

Our results showed clear differences in the gene contents of the mitogenomes studied (Table 4). They all contain genes encoding components of the oxidative-phosphorylation machinery, subunits of the cytochrome c-oxidase complex of ATP synthase, and the cytochrome b subunit. However, the *rps* genes were absent from the *A. chrysogenum* mitogenome. ORFs were present in both *F. oxysporum* and *E. album*. Therefore, the gene contents in Hypocreales are highly conserved.

**Table 4:** Comparison of G + C content (%) of the protein-coding and rRNA genes of mitochondrial genomes of Hypocreales species.

Gene or region	Ach	Fox	Hje	Lsa	Mch	Eal
*cox1*	27.14	32.27	26.02	30.48	32.39	31.61
*cox2*	27.25	28	26.77	28	28.93	34.47
*cox3*	29.38	32.35	30.74	30	30.99	28.76
*cob*	28.41	29.58	28.25	28.6	31.2	26.74
*nad1*	27.69	27.57	25.63	24.64	27.39	25.13
*nad2*	22.8	24.42	24.2	22.28	25	21.7
*nad3*	21.98	23.91	23.43	21.67	27.54	24.52
*nad4*	23.14	25.59	25.71	23.88	25.72	23.18
*nad4L*	22.96	24.07	23.33	24.07	25.93	24.44
*nad5*	25.56	27.51	26.94	25.42	29.32	25.8
*nad6*	23.09	23.07	22.7	19.91	23.16	18.69
*atp6*	27.25	26.72	27.56	25.03	27.35	25.13
*atp8*	20.41	21.09	20.92	23.13	20.41	20.41
*atp9*	31.14	34.22	34.31	32.89	36	32
*orf77*		26.56				23.38
*orf148*						23.71
*rrnS*	35.43	37.67	35.18	35.32	35.34	34.54
*rrnL*	26.99	33.94	31.39	27.43	27.98	31.82
*rps*		21.5	19.02	16.67	19.29	16.22
EmtG	26.54	31.06	27.24	26.53	28.28	25.87

Ach: *Acremonium chrysogenum*; EmtG: entire mitochondrial genome; Fox: *Fusarium oxysporum*; Hje: *Hypocrea jecorina*; Lsa: *Lecanicillium saksenae*; Mch: *Metacordyceps chlamydosporia*

**Figure 3:**
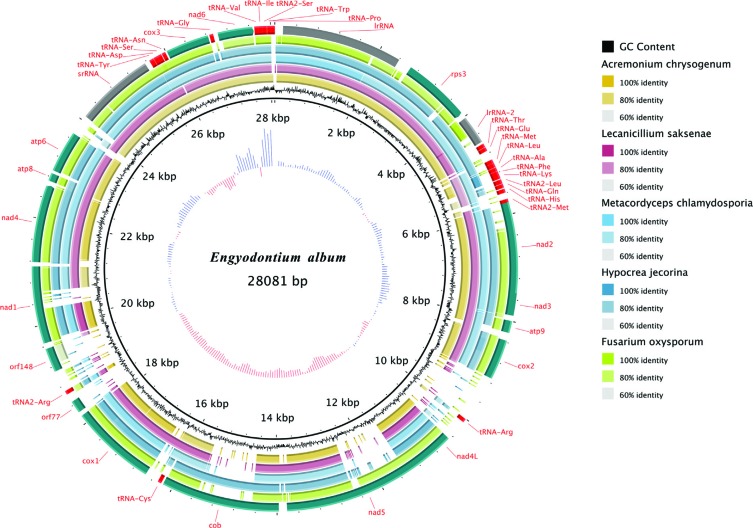
Genome-similarity comparison ring constructed using BRIG software.

Comparison of the Hypocreales mtDNA sequences revealed that they were fairly well conserved, with almost 80% sequence identity in the genomic regions shared with that of *E. album* and only major differences existing in the regions containing the tRNA-Arg (8.8k–12k), *nad5* (11.5k–12.5k), *cob* (14.4k–15.6k), *orf148* and *orf77* (18.1k–19.9k), and *nad1* (20.3k–20.5k) genes. In addition, no gene-module rearrangement occurred in these species, as can be seen in the BRIG map (Figure 3).

### Phylogeny analysis

**Figure 4:**
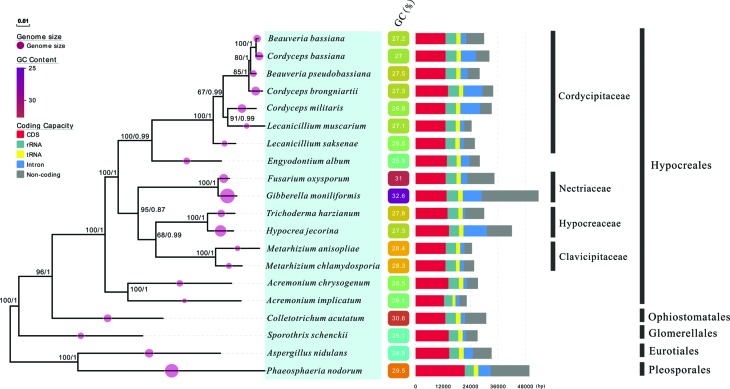
Phylogenetic tree of Ascomycota species. The numbers shown beside the branches indicate ML bootstrap probabilities from 1000 replicates.

**Figure 5:**
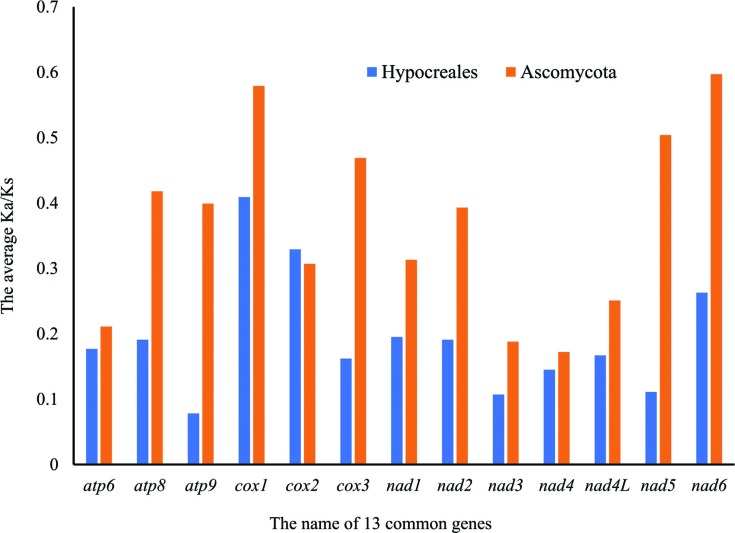
Ka/Ks ratio of pair-wise comparison among the species in the Hypocreales and Ascomycota according to 13 common PCGs.

To investigate the phylogenetic position of *E. album* and the inner relationships of the order Hypocreales, phylogenetic trees were constructed using the nucleotide sequences of 13 PCGs from 20 complete mitochondrial genomes that belong to the Ascomycota division. The phylogenetic trees reconstructed using the ML and Bayes algorithms revealed different clades, which represented five orders, including Hypocreales, Pleosporales, Eurotiales, Glomerellales, and Ophiostomatales (Figure 4). The species in three different families, namely Nectriaceae (*F. oxysporum* and *Gibberella moniliformis*), Hypocreaceae (*H. jecorina* and *Trichoderma harzianum*), and Clavicipitaceae (*M. chlamydosporia* and *Metarhizium anisopliae*), branched in the same clade and then clustered with the *Acremonium implicatum* and *A. chrysogenum* species. The species in the Hypocreales order all clustered within the same clade. *E. album* was located with species in the Cordycipitaceae family with a strong node-supporting value (100% for ML and 1 for Bayes). Examination of the pairwise Ka/Ks ratio for the 13 common PCGs in the Hypocreales and Ascomycota taxa demonstrated that all these genes have undergone purifying selection (Ka/Ks < 1) (Figure 5). Among the species in the Hypocreales order, the Ka/Ks ratio was higher in the *cox1* (0.409), *cox2* (0.329), and *nad6* (0.263) genes than in other genes, while among the species in the Ascomycota division, the most variable genes were *nad6* (0.597), *cox1* (0.579), and *nad5* (0.504).

## Discussion

Many fungi have a significant adverse impact on global human and animal health (Campbell and Johnson, 2013). A particularly important example is the Cordycipitaceae family of fungi (Menzies and Turkington, 2015). *E. album* is a widespread species that poses allergic, pathogenic, or toxic risks to humans and mammals (Siegel and Shadduck, 1990; Goettel *et al.*, 2001; Tucker *et al.*, 2004; Balasingham *et al.*, 2011). Despite advances in sequencing and bioinformatics technologies, only limited characterization of their mitogenomes has been conducted. Here, we sequenced the whole mitochondrial genome of *E. album*, and then compared its genome structure, content, and phylogenetic relationships with other fungal mitogenomes. The mitochondrial genome of *E. album* is a circular DNA molecule of 28,081 bp in length. This size is comparable to that of previously sequenced mitogenomes of members of the Hypocreales order, such as *A. chrysogenum* (27,266 bp) (Eldarov *et al.*, 2015), *L. saksenae* (25,919 bp) (Xin *et al.*, 2017), and *M. chlamydosporia* (25,615 bp) (Ghikas *et al.*, 2006). The average AT content of the *E. album* complete mitogenome is 74.13%, just like the A+T contents reported for *A. Chrysogenum* (74.13%) and *L. saksenae* (74.13%) (Xin *et al.*, 2017). The *E. album* mitogenome gene arrangement is identical to that of other Cordycipitaceae family members, such as *Ophiocordyceps sinensis* (Li *et al.*, 2015), *Beauveria pseudobassiana* (Oh *et al.*, 2015), *Cordyceps militaris* (Sung, 2015), and *Hirsutella minnesotensis* (Zhang *et al.*, 2016). In addition, the PCGs of the *E. album* mt genome were inferred to start with ATG, which is consistent with the arrangement in the mt genomes of other Cordycipitaceae family members (Oh *et al.*, 2015; Sung, 2015). The gene-structure comparison showed that *E. album* has the same gene order and shares homology with the highly conserved mt genomes found within other members of the Hypocreales order. Like other mitogenomes, the rrnS and rrnL genes are located between *atp6* and tRNA-Lys, and between tRNA-Pro and rps, respectively. The GC contents of the *E. album rrnS* and *rrnL* genes are 34.54% and 31.82%, respectively, which is within the range of other Cordycipitaceae mitogenomes (Table 4).

For decades, there has been considerable debate concerning the validity of the taxonomical classification of the *Engyodontium* species. Regarding *E. album*, it was previously included in the *Beauveria* genus. In 1940, this genus was renamed *Tritirachium* and reclassified as a member of the Moniliaceae family. However, *E. album* was later re-assigned to the *Engyodontium* genus (Hoog, 1972). Due to insufficient morphological features, the phylogenetic framework of *Engyodontium* has been little explored, even though the sequences of the 18S and 28S ribosomal RNA genes, the nuclear ribosomal internal transcribed spacer, and the *cox1* gene sequences are available (Seifert, 2009; Schoch *et al.*, 2012). Alternatively, mt genome sequences may provide reliable genetic markers in examining the taxonomic status of *E. album*. Phylogenetic analysis indicated that species in Nectriaceae, Hypocreaceae, Clavicipitaceae, and Cordycipitaceae are well resolved. As a member of the Cordycipitaceae family, *E. album* showed, as expected, a close genetic relationship with the Cordycipitaceae family. This finding was also supported by AT/GC-skew values and sequence differences in PCGs at both the nucleotide and amino acid levels among five representative Hypocreales species. However, no exact data exist yet regarding other lineages of Hypocreales. Therefore, it would be meaningful if a comprehensive phylogeny of Hypocreales is performed in the future, after more mt genome data become available, especially the mitogenome sequences of genera with currently incomplete sequences, such as *Engyodontium* and *Elaphocordyceps*.

In conclusion, the complete nucleotide sequence of the *E. album* mt genome was determined in this study. Comparative analysis showed that the structure, organization, and gene content of *E. album* mtDNA are highly similar to that of species in the Cordycipitaceae family. The availability of the complete mt genome sequence of *E. album* provides novel genetic markers for exploring cryptic/sibling species relating to the Hypocreales order; for preventing infection; and for further studies of the epidemiology, biology, population genetics, and phylogenetic systematics of *E. album*.
